# Protective effect of *Anoectochilus burmannicus* extracts and its active compound, kinsenoside on adipocyte differentiation induced by benzyl butyl phthalate and bisphenol A

**DOI:** 10.1038/s41598-023-30227-5

**Published:** 2023-02-20

**Authors:** Pensiri Buacheen, Jirarat Karinchai, Natchapon Kammasit, Piya Temviriyanukul, Chutikarn Butkinaree, Santi Watthana, Ariyaphong Wongnoppavich, Arisa Imsumran, Pornsiri Pitchakarn

**Affiliations:** 1 Department of Biochemistry, Faculty of Medicine, Chiang Mai University, Chiang Mai, 50200 Thailand; 2 Institute of Nutrition, Mahidol University, Salaya Campus, Nakhon Pathom, 73170 Thailand; 3 Food and Nutrition Academic and Research Cluster, Institute of Nutrition, Mahidol University, Nakhon Pathom, 73170 Thailand; 4 National Omics Center, National Science and Technology Development Agency, Pathum Thani, 12120 Thailand; 5 School of Biology, Institute of Science, Suranaree University of Technology, Nakhon Ratchasima, 30000 Thailand

**Keywords:** Endocrine system and metabolic diseases, Metabolic disorders, Pharmaceutics, Drug discovery and development, Mechanism of action, Natural products, Pharmacology

## Abstract

Benzyl butyl phthalate (BBP) and bisphenol-A (BPA) are obesogens that have been reported to be associated with obesity. Inhibition of their adipogenic activity could decrease the risk of obesity-related metabolic disorders. This study hypothesized that *Anoectochilus burmannicus* ethanolic extract (ABE) which has been previously reported its anti-inflammation property and its known active compound, kinsenoside (Kin) abrogate BBP- and BPA-induced adipogenesis. ABE and Kin markedly suppress both BBP- and BPA-stimulated adipogenesis with different modulations on adipogenic-gene expression including C/EBPα, PPARγ, adiponectin, and leptin in 3T3-L1. BBP induced C/EBPα, adiponectin, and leptin mRNA expressions and slightly increased PPARγ mRNA level, whereas BPA markedly induced PPARγ and adiponectin mRNA levels. ABE significantly decreased the expression of C/EBPα and leptin, but not PPARγ and adiponectin in the BBP-treated cells. In the BPA-treated cells, ABE significantly decreased the mRNA expression of C/EBPα and PPARγ, but not adiponectin and leptin. Interestingly, Kin significantly overcame BBP- and BPA-induced C/EBPα, PPARγ, adiponectin, and leptin expressions. This study first provides evidence to support the health benefits of this plant, especially for people exposed to obesogens. Besides, this finding would encourage the conservation and culture of this orchid for development as an economic plant and healthy food.

## Introduction

Obesity is currently a serious public health issue causing an impaired sense of well-being, impaired quality of life, numerous complications, and increased mortality. It is related to metabolic syndrome and many chronic diseases such as type 2 diabetes mellitus (T2DM), dyslipidemias, high blood pressure (HBP), heart disease, cardiovascular disease (CVD), cancer progression, non-alcoholic fatty liver disease, chronic kidney disease (CKD) and sleep apnea^1^. So, prevention or treatment of obesity would be a strategy to prevent its associated diseases which become serious health problems worldwide. Obesity is characterized by increased fat cell size (hypertrophic obesity) or cell number (hyperplasic obesity) and is caused by intricate interactions between genetic, behavioral, and environmental factors. Although most of the attention has been given to sedentary lifestyles and high-calorie diets as the primary causes, there is growing interest in the involvement of environmental variables.

The increase in rates of obesity is associated with an exponential increase in synthetic chemical production leading to the ‘environmental obesogen’ hypothesis. Chemical obesogens are mainly used in industries and are also found in many daily-life products such as plastic toys, food packaging, plastic bottles, pharmaceuticals, clothing, furniture, food colorings, and pesticides. They can leak into the environment and enter the human body via several routes such as ingestion, inhalation, injection, transdermal contact, and transplacental carriage^2^. Obesogens are “endocrine disrupting compounds” (EDCs) because they affect human metabolism, predispose some people to weight gain, and contribute to the development of obesity and metabolic disorders^3–5^. For example, benzyl butyl phthalate (BBP) and bisphenol-A (BPA) are xeno-estrogen plastic components that are commonly distributed worldwide. Though both have a short half-life, humans are chronically exposed to low doses of these compounds, mainly through modern fast-food, processed/packaged food diets, dust, thermal paper, etc.^6^. BBP, BPA, and their metabolites are therefore detectable in human body fluids, such as blood, urine, saliva, amniotic fluid, and breast milk^7,8^. Previous studies in humans have shown that BBP and BPA levels in the body are associated with obesity, increased waist circumference, and insulin resistance^9,10^. It is also noteworthy that they can accumulate in the adipose tissue and stimulate adipogenesis.

Adipogenesis is a process of differentiation for the change of fibroblast-like preadipocyte to mature adipocyte^11^. In brief, it is a two-step developmental process in which an undifferentiated mesenchymal cell differentiates into a preadipocyte, which then undergoes a secondary differentiation step to become a lipid-filled adipocyte. The adipogenesis is activated by some transcriptional factors, for example, C/EBPs (CCAAT-enhancer-binding proteins) and PPARs (Peroxisome proliferator-activated receptor)^12^. Besides, some adipokines such as adiponectin and leptin participate in adipogenesis to alter the cellular composition, cell number, and structure leading to mature adipocyte formation^3,4,13^. During the entire differentiation, C/EBPβ and C/EBPδ are key early regulators of adipogenesis since they promote the expression of other key adipogenic transcription factors including C/EBPα and PPAR to regulate a wide range of gene expression for the development of mature adipocytes^14^. Interestingly, in vitro studies found BBP and BPA could promote adipogenesis in 3T3-L1 preadipocytes via the PPARγ-C/EBPα pathway^15,16^. The prevention or inhibition of obesogen-induced adipose formation by natural products can be vital in the fight against the growing incidence of obesity.

*Anoectochilus burmannicus* (*A. burmannicus*), a jewel orchid, is a plant in the *Anoectochilus* genus which has been used in traditional medicine, for example, clearing heat, cooling blood, removing dampness, and detoxification^17–23^. Many previous studies have reported its activity against inflammation, oxidative stress, and insulin resistance^24,25^. Besides, *A. burmannicus* extract exerted anti-obesity by inhibiting lipid droplet accumulation and decreasing the number of differentiated adipocytes^26^. In *Anoectochilus* species, kinsenoside (Kin) is known as a major bioactive component that could reduce the triglyceride level and body weight of high-fat diet rats^27,28^. Therefore, we hypothesized that the *A. burmannicus* extract also contains Kin and they both can abrogate the adipogenic effect of BBP and BPA.

The present study aimed to investigate whether *A. burmannicus* extract and Kin can reduce or inhibit obesogen-induced adipogenesis and adipogenic gene expression in the 3T3-L1 adipocytes. The knowledge from this study would support the development of this orchid as an alternative anti-obesity agent and can promote this orchid to be an economic and healthy plant/ food in the future.

## Results

### BBP and BPA significantly induce adipogenesis of 3T3-L1 cells

**Figure 1:**
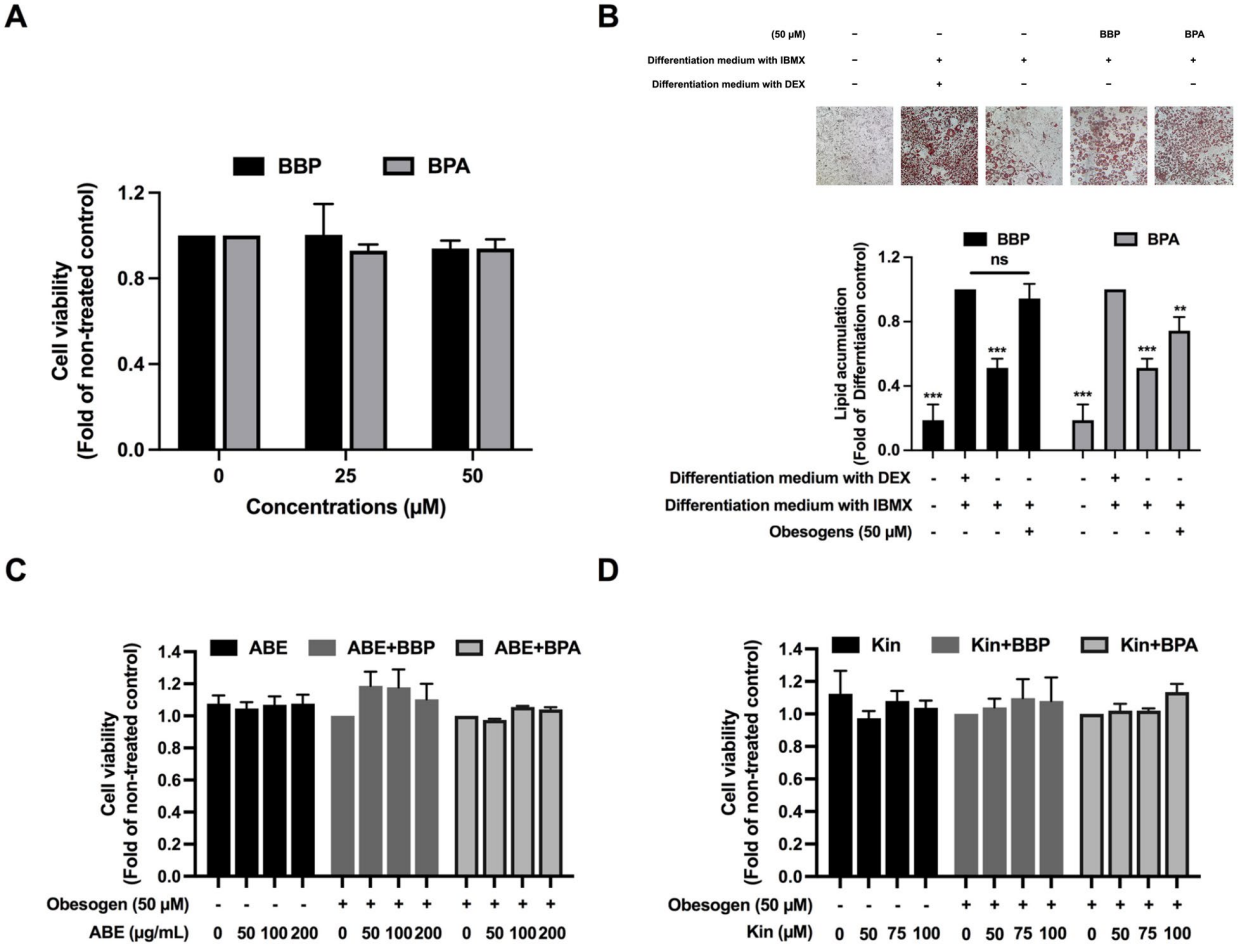
Cytotoxicity of BBP and BPA on 3T3-L1 adipocyte during adipocyte differentiation tested by SRB assay (**A**), BBP and BPA induced lipid accumulation in adipocyte (**B**). Lipid droplet staining using Oil red O dye and the level of lipid accumulation obtained by spectrophotometry of the dye dissolved, Cytotoxicity of ABE (**C**) and Kin (**D**) in the absence or presence of BBP or BPA investigated by SRB assay. The data indicated as mean ± SD of three independent experiments **p < 0.01 and ***p < 0.001, compared to differentiation control (**B**).

Treatment of BBP or BPA during adipocyte transformation (Day 1–12), at 100 μM slightly decreased cell viability when compared with the non-treated control (data not shown), while at 25 and 50 μM did not affect the cell viability (Fig. 1A). Therefore, the concentration at 50 μM of BBP and BPA was used for further experiments.

The adipogenic effects of BBP and BPA were determined by measuring the content of intracellular lipid droplets in 3T3-L1 adipocytes using an oil-red-O assay. It was found that BBP and BPA were able to stimulate adipocyte differentiation in the absence of dexamethasone (DEX), a strong adipogenesis inducer. BBP and BPA (50 μM) significantly induced adipogenesis at approximately 40% (*p* < 0.001) and 20% (*p* < 0.01), respectively as compared to the non-treated control (Fig. 1B). Besides, the increase of lipid droplets in the BBP- and BPA-treated cells was remarkable under microscopic observation compared to the non-treated control. The dose of 50 μM was then subjected to the next experiments to determine whether ABE and Kin inhibit adipocyte differentiation induced by these obesogens.

### ABE and Kin abrogate BBP- and BPA-induced adipogenesis of 3T3-L1 cells

As a quality control of the plant sample and the extraction, the total phenolic content of ABE was 8.61 ± 0.21 mg GAE/g extract which was equivalent to the ABE extract used in our previous study^25^. Before determining the effect of ABE and Kin on adipogenesis induced by BBP or BPA (50 μM) in the 3T3-L1 cells, the cytotoxic effect of the co-treatments with ABE or Kin and each obesogen was first evaluated during transformation (Days 1–12) using SRB assay. It was found that ABE at up to 200 μg/mL and Kin at up to 100 μM did not cause any toxicity in the 3T3-L1 cells treated with BBP or BPA (Fig. 1C,D). Therefore, ABE and Kin at 200 μg/mL and 100 μM, respectively, were subjected to the next experiments.

**Figure 2:**
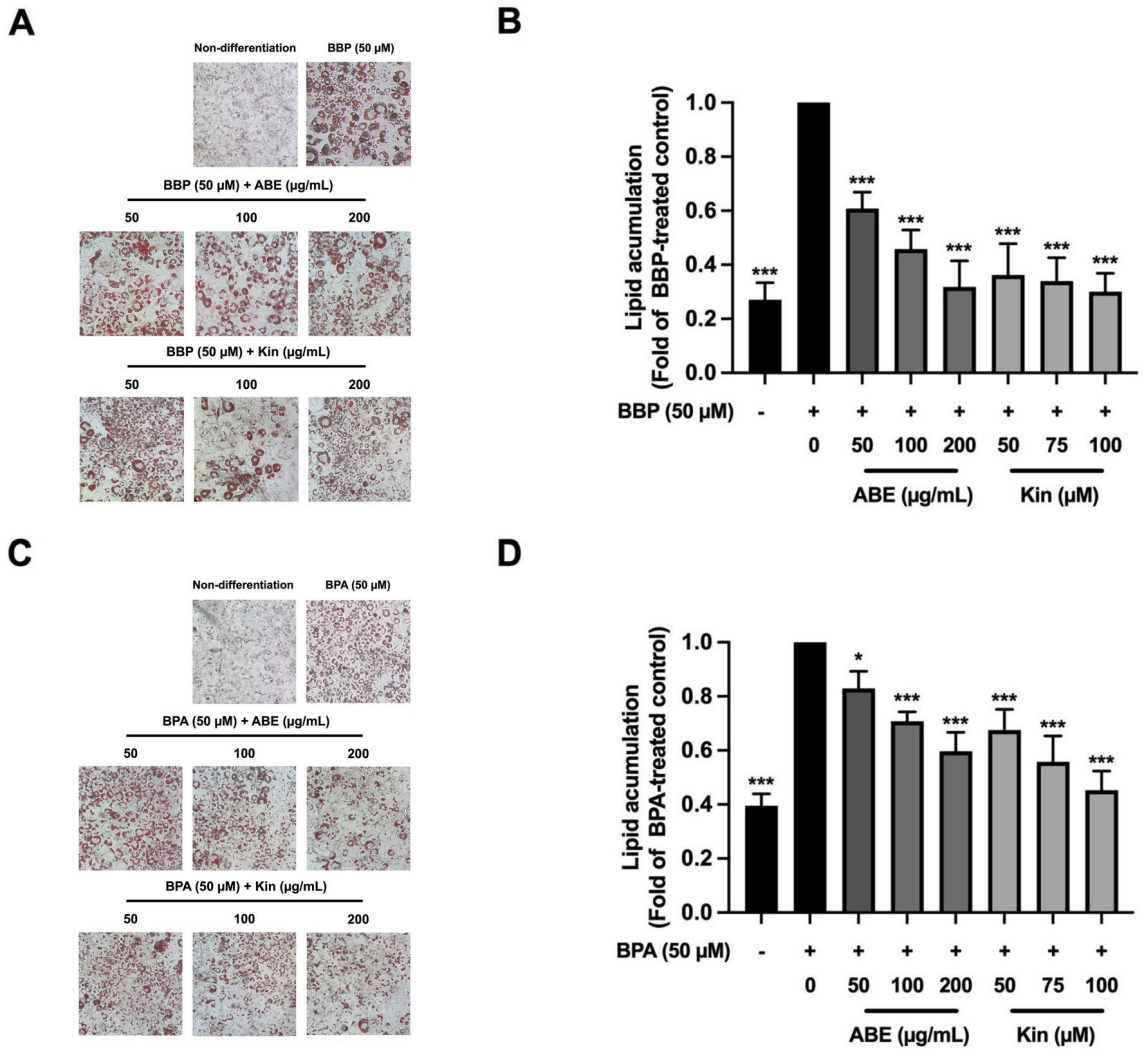
The effect of ABE or Kin on obesogen-induced lipid accumulation in 3T3-L1 cells determined by Oil-red-O staining assay. Intracellular lipid droplet (**A**), and the level of lipid accumulation (**B**) of ABE- or Kin-treated cells in the presence of BBP (50 µM). Intracellular lipid droplet (**C**), and the level of lipid accumulation (**D**) of ABE- or Kin-treated cells in the presence of BPA (50 µM). The data indicated as mean ± SD of three independent experiments *p < 0.05 and ***p < 0.001, compared to obesogen-treated control.

The 3T3-L1 adipocyte was treated with BBP or BPA (50 μM) and the nontoxic doses of ABE (0–200 μg/mL) or Kin (0–100 μM) during differentiation (Days 1–12). Intracellular lipid accumulation was detected by an Oil-Red-O staining assay to confirm adipocyte maturation^29^. The result showed that both ABE and Kin significantly decreased the lipid accumulation of the cells treated with BBP (Fig. 2A,B**)** or BPA (Fig. 2C,D). Since BBP is strong but BPA is a moderate obesogen, the inhibitory effects of ABE and Kin were lesser in the BPA-treated cells than the BBP-treated cells. Nonetheless, these results could suggest that ABE and Kin diminish the obesogenic effects of BBP and BPA via inhibition of adipogenesis which is a critical process of obesity development.

### ABE and Kin reduce expression of adipogenic genes, C/EBPα and PPARγ in BBP- or BPA-treated 3T3-L1 adipocytes

**Figure 3:**
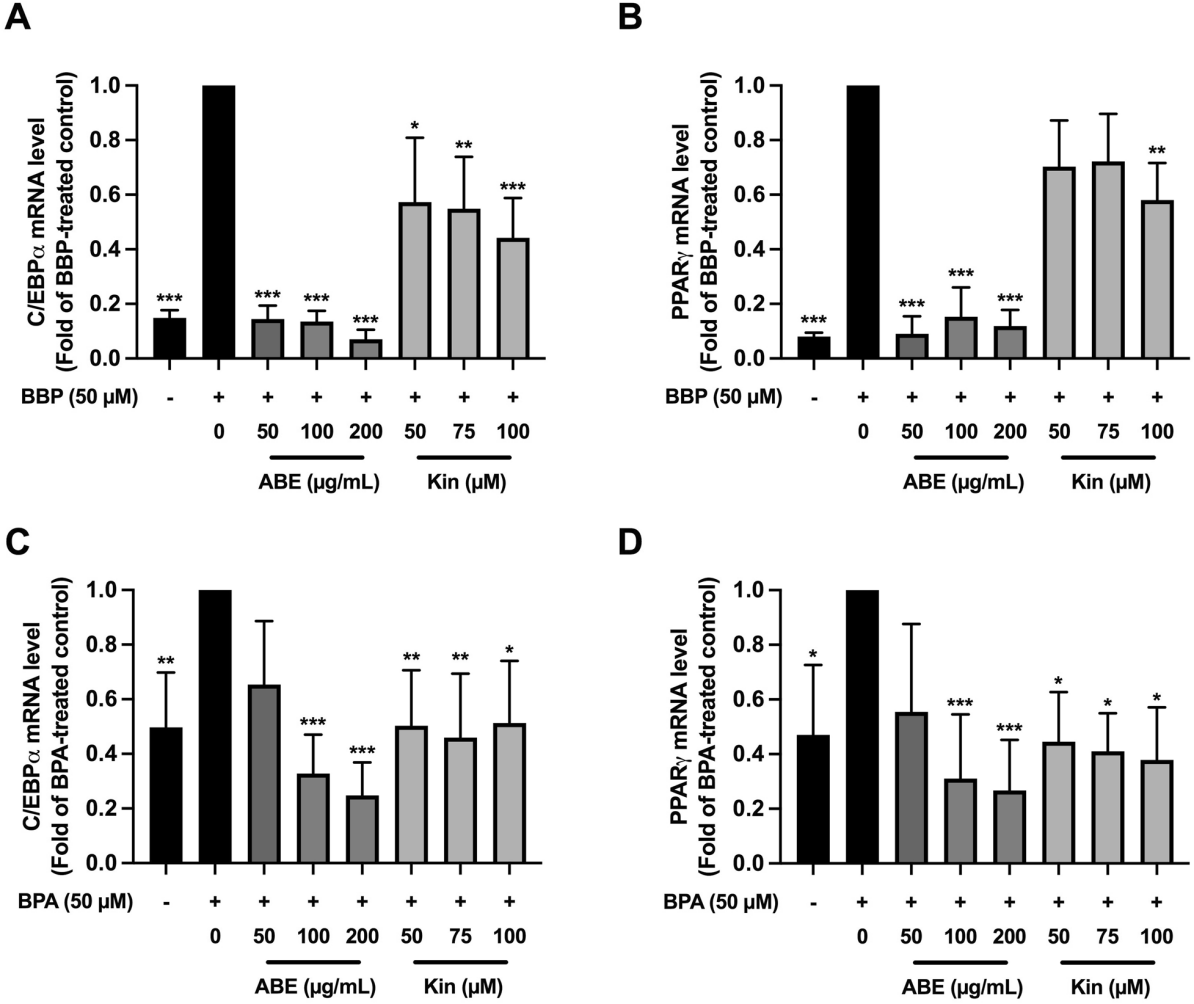
The effect of ABE or Kin on obesogen-induced adipogenic gene expression in 3T3-L1 cells determined by RT-qPCR. C/EBPα (**A**,**C**) and PPARγ (**B**,**D**) mRNA level in the cells treated with ABE or Kin in the presence of BBP or BPA (50 µM). The data indicated as mean ± SD of three independent experiments *p < 0.05, *p < 0.01 and ***p < 0.001, compared to obesogen-treated control.

C/EBPα and PPARγ are known to be important key regulators for adipocyte formation^12^. The inhibition of BBP- and BPA-induced adipogenesis by ABE and Kin is likely involved with the down-regulation of adipogenic gene expression. Thus, the expression of C/EBPα and PPARγ was quantitatively determined by RT-qPCR in the 3T3-L1 adipocyte treated with each obesogen and ABE or Kin. As shown in Fig. 3, treatment of BBP or BPA (days 1–12) could significantly increase the mRNA levels of both C/EBPα and PPARγ, yet BBP showed stronger effects than BPA. When compared to the non-treated control, BBP and BPA significantly stimulated the mRNA expressions of C/EBPα by 86% (*p* < 0.001) and 51% (*p* < 0.01), respectively, and that of PPARγ by 92% (*p* < 0.001) and 53% (*p* < 0.05), respectively. Interestingly, the induction of adipogenesis gene expression by BBP or BPA could be blocked by ABE and Kin. ABE (50, 100, 200 μg/mL) strongly and significantly inhibited C/EBPα (by 85%, 86%, 91%, *p* < 0.001) and PPARγ (by 90%, 84%, 88%, *p* < 0.001) mRNA expressions induced by BBP (Fig. 3A,B). On the other hand, Kin at 50, 75, and 100 μM significantly reduced the mRNA level of C/EBPα by 42% (*p* < *0.5*), 45% (*p* < *0.01*), 55% (*p* < 0.001)) while Kin only at high dose (100 μM) significantly decreased PPARγ mRNA level in the BBP-treated cells (Fig. 3A,B). In the combination treatment between BPA and ABE or Kin in 3T3-L1 cells, we found that ABE at 100 and 200 μg/mL significantly decreased the mRNA levels of C/EBPα (by 70%, 75%, *p* < 0.001) and PPARγ (by 70%, 74%, *p* < 0.001) as shown in Fig. 3C,D. However, high dose of Kin (100 μM) significantly inhibited BPA-induced C/EBPα and PPARγ mRNA expressions by 50% (*p* < 0.05) and 63% (*p* < 0.05), respectively (Fig. 3C,D). These results suggested that ABE and Kin abrogated BBP- and BPA-induced adipogenesis by down-regulation of C/EBPα and PPARγ mRNA expressions.

### Alteration of ABE and Kin on adiponectin and leptin gene expressions in the BBP- and BPA-treated 3T3-L1 adipocytes

**Figure 4:**
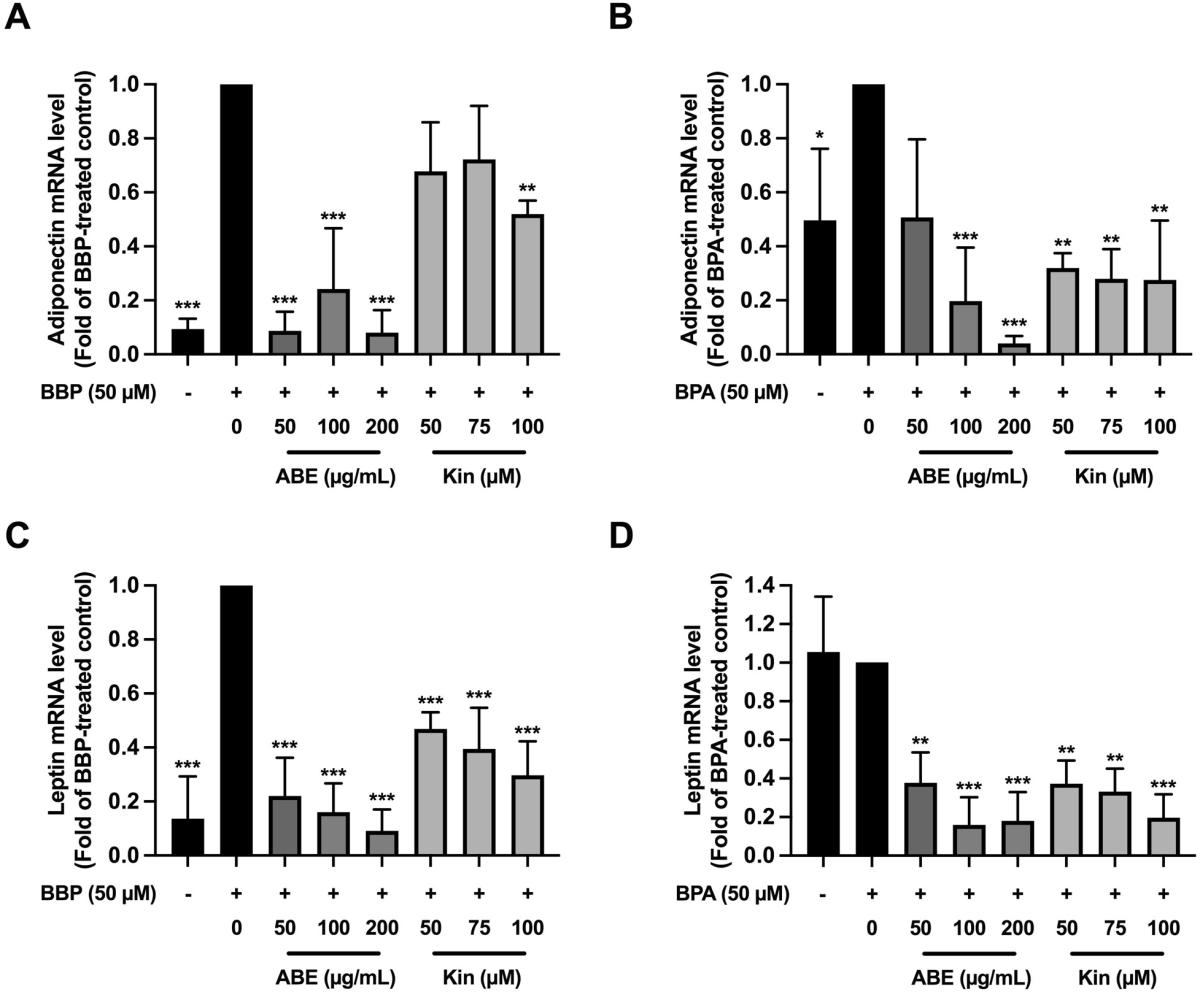
The effect of ABE or Kin on obesogen-induced adipokine gene expression in 3T3-L1 cells determined by RT-qPCR. Adiponectin mRNA level in BBP- (**A**) and BPA- (**B**) treated cells in the presence of ABE or Kin. Leptin mRNA level-induced in BBP- (**A**) and BPA- (**B**) treated cells in the presence of ABE or Kin. The data indicated as mean ± SD of three independent experiments *p < 0.05, **p < 0.01 and ***p < 0.001, compared to obesogen-treated control.

Adiponectin and leptin are adipokines secreted from mature adipocytes and they are used as biomarkers of adipocyte maturation or formation. Next, we also investigated the effect of ABE and Kin on adiponectin and leptin mRNA expressions in the BBP- and BPA-treated cells. Both BBP and BPA significantly upregulated while cotreatment of the cells with ABE or Kin markedly downregulated the adiponectin expression (Fig. 4). In the BBP-treated cells (Fig. 4A), adiponectin mRNA level was significantly decreased by 50, 100, and 200 μg/mL of ABE at approximately 92% (*p* < 0.001), 76% (*p* < 0.001), and 92% (*p* < 0.001), respectively as well as by 50 and 100 μM of Kin at approximately 32% (*p* < 0.01) and 48% (*p* < 0.01), respectively. In the BPA-treated cells (Fig. 4B), adiponectin mRNA level was significantly suppressed by 100 and 200 μg/mL of ABE at approximately 80% (*p* < 0.001), and 90% (*p* < 0.001), respectively. Likewise, the adiponectin mRNA was reduced by 50, 75, and 100 μM of Kin at approximately 70% (*p* < 0.01), 73% (*p* < 0.01), and 73% (*p* < 0.01), respectively.

As shown in Fig. 4C,D, BBP and BPA showed different effects since only BBP significantly induced the expression of leptin. In the BBP-treated cells, ABE (50, 100, and 200 μg/mL) significantly suppressed the leptin mRNA level at approximately 74% (*p* < 0.001), 84% (*p* < 0.001), and 90% (*p* < 0.001), respectively. Besides, Kin (50, 75, and 100 μM) significantly suppressed the leptin expression at approximately 54% (*p* < 0.001), 61% (*p* < 0.001), and 71% (*p* < 0.001), respectively (Fig. 4C). Although the induction of leptin mRNA expression by BPA was not significant, it was significantly suppressed by 50, 100, and 200 μg/mL of ABE at approximately 63% (*p* < 0.01), 84% (*p* < 0.001), and 82% (*p* < 0.001), respectively as well as by 50, 75, and 100 μM of Kin at approximately 53% (*p* < 0.01), 55% (*p* < 0.01), and 65% (*p* < 0.001), respectively (Fig. 4D).

Taken together the results confirmed that ABE and Kin could delay or inhibit the BBP- and BPA- stimulated adipocyte maturation and lipogenesis via the suppression of adipogenesis-related gene expression.

## Discussion

Obesogens are a group of chemicals or compounds that interfere with lipid metabolism, and in some cases can lead to obesity^30^. Interestingly, the chemical obesogens are capable of inducing lipid accumulation which is a hallmark of adipogenesis in adipocytes^31^. Previous studies showed that enhanced adipogenesis during preadipocyte differentiation is a potential mechanism underlying the weight gain which is associated with the exposure of phthalates^32–34^. Both BBP and BPA are lipophilic agents that can pass the blood–brain barrier and accumulate in the adipose tissue. They have been shown to enhance preadipocyte differentiation and lipid accumulation in adipocytes via the induction of transcription factors and adipocyte-specific gene expressions including C/EBPα, PPARγ, adiponectin, and leptin^15,35^. Additionally, in vivo study found that HFD-fed male mice exposed to moderate concentration (3 mg/kg bw/day) of BBP had higher body weight compared to the control group due to the significant increase of liver and adipose tissue masses^36^. Besides, maternal-BPA exposure could induce offspring adiposity and hypertrophic adipocytes and increase the expression of pro-adipogenic and lipogenic factors in Sprague Dawley rats^37,38^. This information can be evidence to support the relationship between obesity development and obesogen exposures. Avoiding exposure or suppressing the activity of BBP and BPA may help to prevent the expansion of adipose tissue that causes obesity.

As a suitable screening assay for anti-adipogenic agents against BBP and BPA, we have modified the adipogenesis induction protocol from the previous study^39^. First, we showed that 50 µM BBP was not toxic to the adipocyte whereas 50 µM BPA showed slight cytotoxicity (< IC_20_). Both BBP and BPA at a dose of up to 100 µM were also shown to be non-toxic to the cells^40^. Second, our results showed that BBP and BPA themselves can activate adipogenesis without having an adipogenesis stimulator, DEX. Yet, adipocyte differentiation by BBP and BPA requires a longer time. Thus, DEX must be replaced by the obesogens to evaluate their adipogenesis stimulation in concomitant with IBMX which acts as a co-activator of the C/EBPα and PPARγ pathway^41^. Similar to our study, BBP and BPA could stimulate adipocyte differentiation in the DEX-free induction medium^39^. However, the concentration of DEX could be adjusted in the study of obesogen-induced adipogenesis since Sakuma et al. 2017^42^ reported a treatment model using a lower concentration of DEX (less than 1 µM). Like DEX, BBP and BPA can stimulate glucocorticoid receptors which in turn lead to C/EBPα and PPARγ activations and subsequently adipocyte formation^42^. Our results also confirmed that BBP and BPA can induce C/EBPα and PPARγ mRNA expressions in 3T3-L1 adipocytes. Finally, we found that BBP and BPA were acquired in both the initiation and termination steps of adipocyte formation. Adiponectin and leptin are adipokines that indicate mature adipocyte or adipocyte formation. Interestingly, while both BBP and BPA stimulated the expression of adiponectin, only BBP induced that of leptin. It is noteworthy that BBP exerted higher inducing adipogenesis activity than BPA. The result is in agreement with the previous report indicating BBP as a strong obesogen and BPA as a weak obesogen^43^. The inhibition of the adipogenic activity of obesogens might be a promising way to prevent obesity and decrease the risk of its associated complications.

In the present study, we are interested in the effect of *Anoectochilus burmannicus* extract (ABE) and its bioactive compound to prevent BBP-, BPA-induced adipocyte formation because ABE has been previously shown many biological activities including anti-inflammation, anti-insulin resistance, and anti-DEX-induced adipocyte formation^24–26^. As a major active component, kinsenoside (Kin) isolated from *Anoectochilus* sp., including *A*. *burmannicus* exhibits several biological and pharmacological activities, including hepatoprotective, anti-hyperglycemic, anti-hyperlipidemic, and anti-inflammatory effects^44,45^. Our previous study also confirmed that this compound in ABE possesses anti-inflammatory activity^25^. Herein, we were able to show that both ABE and Kin markedly suppress the BBP- or BPA- stimulated adipocyte formation. The treatment of ABE (50, 100, 200 µg/mL) and Kin (50, 75, 100 µM) during adipogenesis (12 days) significantly reduced the lipid accumulation stimulated by BBP or BPA. The inhibition of either activity or expression of adipogenic genes including C/EBPα, PPARγ, adiponectin, and leptin could delay adipogenesis. As expected, ABE and Kin significantly decreased the expression of C/EBPα, PPARγ, adiponectin, and leptin in the BBP- or BPA-treated adipocytes. Similar to our observation, Satoru et al.^42^ reported that curcumin reduced TG level, the number of Oil Red O-stained cells, and the mRNA expression level of PPARγ, C/EBPα, adiponectin, and tumor necrosis factor-α (TNFα) leading to inhibition of BBP-induced adipogenesis. The alteration of adipogenesis- and lipogenesis-related proteins by ABE and Kin in the BBP- or BPA-treated adipocytes must be further investigated either by proteomic analysis or Western blotting.

In terms of active phytochemicals, the ABE used in the present study has phenolic content of approximately 6.96 ± 0.28 mg GAE/g extract which is close to the content found in the ABE in our previous study^25^. The amount of Kin in ABE is approximately 250 µM/mg extract which is similar to that found in *A. formosanus* aqueous extract^46^. The concentration of Kin (50 µM) used in the present study is paralleled with its content detected in 200 µg/mL ABE. Unexpectedly, it was found that 200 µg/mL ABE exhibits higher effectiveness than that 50 µM Kin. Probably, ABE may contain other active compounds besides Kin which contribute to the inhibitory effect. The unknown active compound(s) in ABE needs further analysis by bioassay-guided study followed by LC–MS/MS analysis.

## Conclusion

In summary, *Anoectochilus burmannicus* extract and kinsenoside abrogate the obesogen (BBP and BPA)-stimulated adipocyte formation by down-regulating the adipogenic gene expressions including C/EBPα, PPARγ, adiponectin, and leptin leading to the decrease of lipid accumulation. The information from this study would raise attention to phytochemicals as anti-chemical-induced obesity and promote the conservation and culture of *Anoectochilus burmannicus*. For the development of ABE as a functional food or ingredient, its active compounds, exact biological mechanisms, inhibitory effects in vivo*,* and safety must be further investigated.

## Materials and methods

All methods were performed in accordance with the relevant guidelines and regulations.

### Chemicals

Benzyl butyl phthalate (BBP), bisphenol A (BPA), dexamethasone (DEX), and 3-isobutyl-1-methylxanthine (IBMX) were purchased from Sigma (Darmstadt, Germany). Kinsenoside was purchased from Biopurify Phytochemicals Ltd. (Sichuan, China), and all the primers for RT-qPCR were purchased from (Bio Basic, Canada).

### Plant sample and the extraction

The plant collection was conducted following the guidelines and regulations of the legislation. *A. burmannicus* sample was tissue cultivated and obtained from the Queen Sirikit Botanic Garden, Chiang Mai, Thailand. The sample was identified by Dr. Santi Watthana (taxonomist), School of Biology, Institute of Science, Suranaree University of Technology, Nakhon Ratchasima, Thailand. The plant sample was deposited in the Queen Sirikit Botanic Garden, Chiang Mai, Thailand. The herbarium voucher specimen is S. Watthana 4494 (QBG). The ethanolic extract (ABE) was prepared as previously described^25^. The extract was filtered and freeze-dried by lyophilization to obtain ethanolic extract powder. The yield of ABE was about 25% (w/w). The ABE powder was kept at −20 °C until used.

### Determination of phenolic content

Total phenolic content was measured according to the previous study with modifications^25^. Different concentrations of the extract were prepared in 120 µL DMSO and then mixed with 80 µL of 10% equivalent Folin–Ciocalteu reagent. The mixture was allowed to stand at room temperature for three minutes, and then 100 μL of 7.5% (W/V) sodium bicarbonate was added to the mixture for 30 min in the dark. Next, the absorbance reading at 765 nm was measured using a spectrophotometer. Acidified ethanol was used as the blank. Total phenolic content was expressed as mg gallic acid equivalent (GAE) /g extract.

### 3T3-L1 cell culture

The 3T3-L1 pre-adipocyte was obtained from American Type Culture Collection, (ATCC, USA) and cultured in Dulbecco's Modified Eagle Medium (DMEM) (Gibco™, Fisher Scientific UK) with l-glutamine supplemented with 10% heat-inactivated calf serum (CS, Gibco™, Fisher Scientific UK) and 1% penicillin/streptomycin solution and maintained at 37 °C in a 5% CO_2_ humidified atmosphere (CO_2_ incubator, Thermo Scientific) and sub-cultured every three days.

### 3T3-L1 differentiation induced by BBP and BPA and the treatment of ABE and kin

The 3T3-L1 pre-adipocyte was differentiated to become a mature adipocyte using three types of adipocyte transformation media including an induction medium, a differentiation medium, and a maturation medium as previously described^47^ with slight modification. The 3T3-L1 pre-adipocyte was cultured to 100% confluence in 10% calf serum DMEM (CS medium), as of day zero. After post-confluence, the cells were incubated with the induction medium (DMEM with 1 μM DEX (dexamethasone), 0.5 mM IBMX (3-isobutyl-1-methylxanthine), 167 nM insulin, and 10% fetal bovine serum (FBS, HyClone Sera. Standard FBS, US Origin)) for three days (Days 1–3). After that, cells were then cultured in the differentiation medium (DMEM containing 167 nM insulin, and 10% FBS) for three days (Days 4–6). Finally, the maturation medium (DMEM containing 167 nM insulin and 10% FBS) was used to induce cellular lipid accumulation for six days (Days 7–12). The adipocyte formation was evaluated by measuring lipid accumulation in the cells using Oil red O staining. To examine the effect of BBP and BPA on adipogenesis, the cells were treated with 25, 50 µM BBP or BPA during the adipocyte transformation (Days 1–12) in the absence of DEX in the initiation step.

To determine the effect of ABE and Kin on the adipogenesis stimulated by BBP or BPA. Three different concentrations of ABE (50, 100, 200 µg/mL) or Kin (50, 75, 100 µM) were added during the adipocyte transformation process (Days 1–12, in the absence of DEX). Cells cultured with the vehicle (0.5% DMSO) in all three types of transformation media were set as the control (BBP- or BPA-treated cells).

### Determination of cytotoxicity in the mature 3T3-L1 adipocyte

BBP, BPA, (25, 50, 100 µM) and a combination of BBP or BPA with ABE or Kin were tested for their cytotoxicity on the mature 3T3-L1 adipocyte by SRB assay to obtain their non-toxic concentration for further experiments. Our previous study found that the cell viability was not affected by ABE (up to 200 µg/mL) and Kin (up to 100 µM)^25^. Briefly, ABE (50, 100, 200 µg/mL) or Kin (50, 75, 100 µM) was added during the adipocyte transformation process (Days 1–12, in the absence of DEX). Cells cultured with the vehicle (0.5% DMSO) in all three types of transformation medium were set as the untreated control (BBP- or BPA-treated cells). The cells were then fixed and subjected to sulforhodamine (SRB) assay according to the previous protocol^48^. The absorbance was spectrophotometrically measured at 510 nm (Gen5, BioTek), and the cell viability was calculated as the ratio of the treatment versus control.

### Determination of lipid accumulation in BBP- or BPA-induced 3T3-L1 adipocyte by Oil-Red-O assay

After the treatment described in “Alteration of ABE and Kin on adiponectin and leptin gene expressions in the BBP- and BPA-treated 3T3-L1 adipocytes” section, the cells were fixed in fresh 4% paraformaldehyde, and the lipid droplets were stained with Oil-Red-O diluted in 100% 2-isopropanol, as previously described^49^. The stained-intracellular lipid droplets were dissolved in 100% 2-isopropanol and the absorbance was measured by spectrometry at 520 nm (Gen5, BioTek). The experiment was performed in triplicate and repeated three times independently.

### Determination of adipogenesis-related gene expression by reverse transcription-quantitative polymerase chain reaction (RT-qPCR)

After the treatment (Day 12) described in “Alteration of ABE and Kin on adiponectin and leptin gene expressions in the BBP- and BPA-treated 3T3-L1 adipocytes” section, the cells were collected and total RNAs were isolated by TRIZol reagent (Invitrogen). The cDNA was synthesized from 1 μg total RNA using RevertAid RT Reverse Transcription Kit (Thermo Fisher Scientific, USA). Quantitative PCR was further conducted using a synthesized cDNA with SensiFAST™ SYBR Lo-ROX Kit (Bioline, United Kingdom). The amplification conditions were initial denaturation at 95 °C for ten minutes, followed by 40 cycles of denaturation at 95 °C for 30 s, annealing at 60 °C for 30 s and elongation at 72 °C for 30 s. Melt-curve analysis was performed to confirm that the signal was that of the expected amplification product. The sequences of oligonucleotide primers were used detection of PPARγ (sense, 5′- TCCGCTGATGCACTGCCTAT-3′; antisense, 5′-GGAATGCGAGTGGTCTTCCA-3′), CEBPα (sense, 5′- GCAAAGCCAAGAAGTCGGTG-3′; antisense, 5′-TCACTGGTCAACTCCAGCAC-3′), Adiponectin (sense, 5′- ATCTGGAGGTGGGAGACCAA-3′; antisense, 5′- GGGCTATGGGTAGTTGCAGT-3′), Leptin (sense, 5′-AAGGGGCTTGGGTTTTTCCA-3′; 5′- CAGACAGAGCTGAGCACGAA-3′) and β-actin (sense, 5′-TGGTGGGAATGGGTCAGAAG-3′; antisense, 5′- TGTAGAAGGTGTGGTGCCAG-3′). The levels of target cDNAs were normalized by β-actin expression and then calculated as a relative expression to the BBP- or BPA-treated alone.

### Statistical analysis

All values were given as mean ± standard derivation (X̄ ± SD) from triplicate samples of two or three independent experiments. Overall differences among the treatment groups were determined using One-way analysis of variance (ANOVA) with the post-hoc Turkey’s test by Prism 9.0 software or the Student's t-test. *p* < 0.05 is regarded as significant.

## Data Availability

The datasets used and/or analysed during the current study available from the corresponding author on reasonable request.
