# Correction: Protective effect of *Anoectochilus burmannicus* extracts and its active compound, kinsenoside on adipocyte differentiation induced by benzyl butyl phthalate and bisphenol A

**DOI:** 10.1038/s41598-025-30577-2

**Published:** 2025-12-03

**Authors:** Pensiri Buacheen, Jirarat Karinchai, Natchapon Kammasit, Piya Temviriyanukul, Chutikarn Butkinaree, Santi Watthana, Ariyaphong Wongnoppavich, Arisa Imsumran, Pornsiri Pitchakarn

**Affiliations:** 1 Department of Biochemistry, Faculty of Medicine, Chiang Mai University, Chiang Mai, 50200 Thailand; 2 Institute of Nutrition, Mahidol University, Salaya Campus, Nakhon Pathom, 73170 Thailand; 3 Food and Nutrition Academic and Research Cluster, Institute of Nutrition, Mahidol University, Nakhon Pathom, 73170 Thailand; 4 National Omics Center, National Science and Technology Development Agency, Pathum Thani, 12120 Thailand; 5 School of Biology, Institute of Science, Suranaree University of Technology, Nakhon Ratchasima, 30000 Thailand

Correction to: *Scientific Reports* 10.1038/s41598-023-30227-5, published online 20 February 2023

The original version of this Article contained an error in Figure 1, panel B, where the images representing the “BPA” and “BBP” groups were swapped. The original Figure 1 and accompanying legend appear below.

**Fig. 1:**
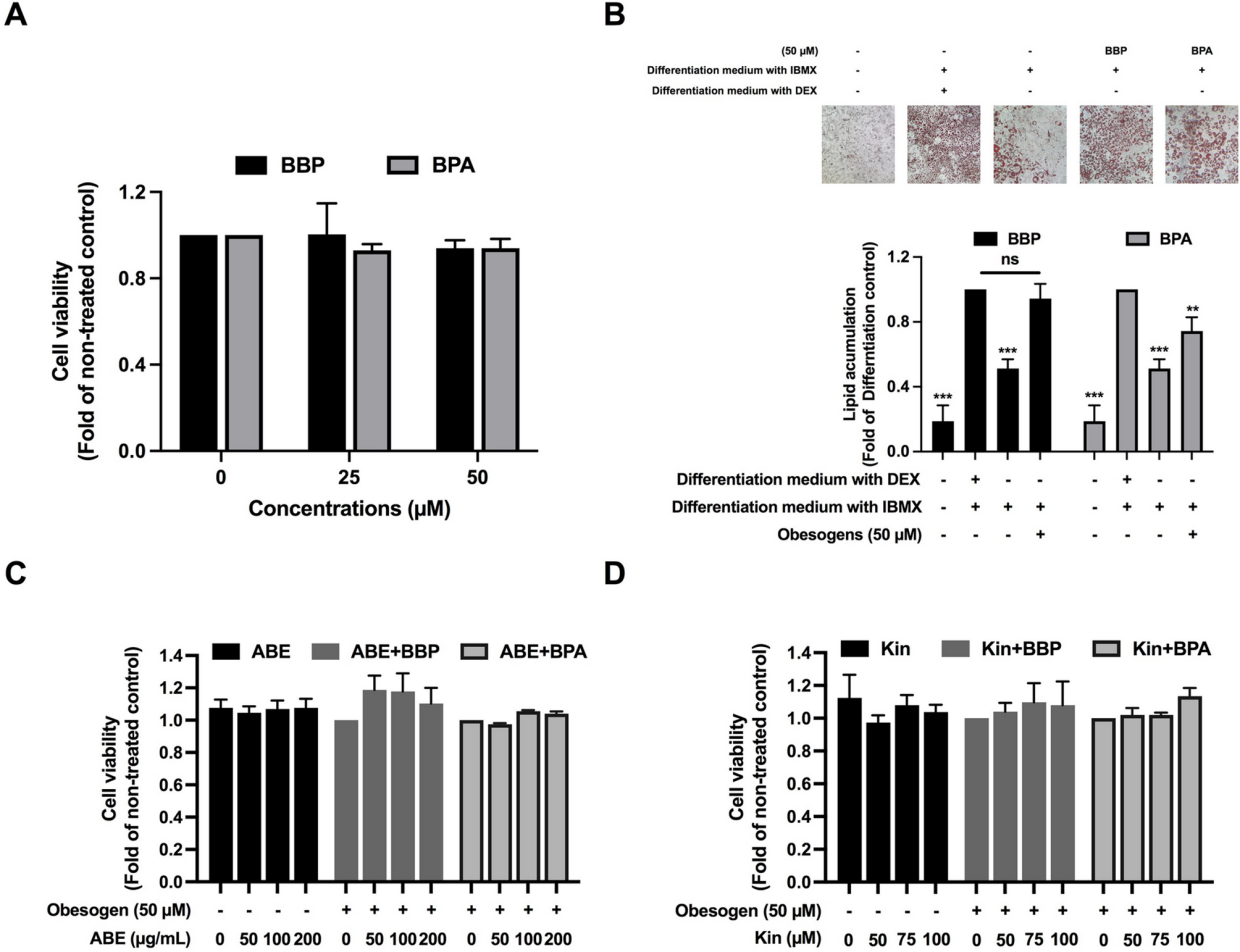
Cytotoxicity of BBP and BPA on 3T3-L1 adipocyte during adipocyte differentiation tested by SRB assay (**A**), BBP and BPA induced lipid accumulation in adipocyte (**B**). Lipid droplet staining using Oil red O dye and the level of lipid accumulation obtained by spectrophotometry of the dye dissolved, Cytotoxicity of ABE (**C**) and Kin (**D**) in the absence or presence of BBP or BPA investigated by SRB assay. The data indicated as mean ± SD of three independent experiments **p < 0.01 and ***p < 0.001, compared to differentiation control (**B**).

In addition, in Figure 2, the image for the “BBP (50 μM)” group in panel A and that for “BPA (50 μM)” group in panel C were incorrect. The original Figure 2 and accompanying legend appear below.

**Fig. 2:**
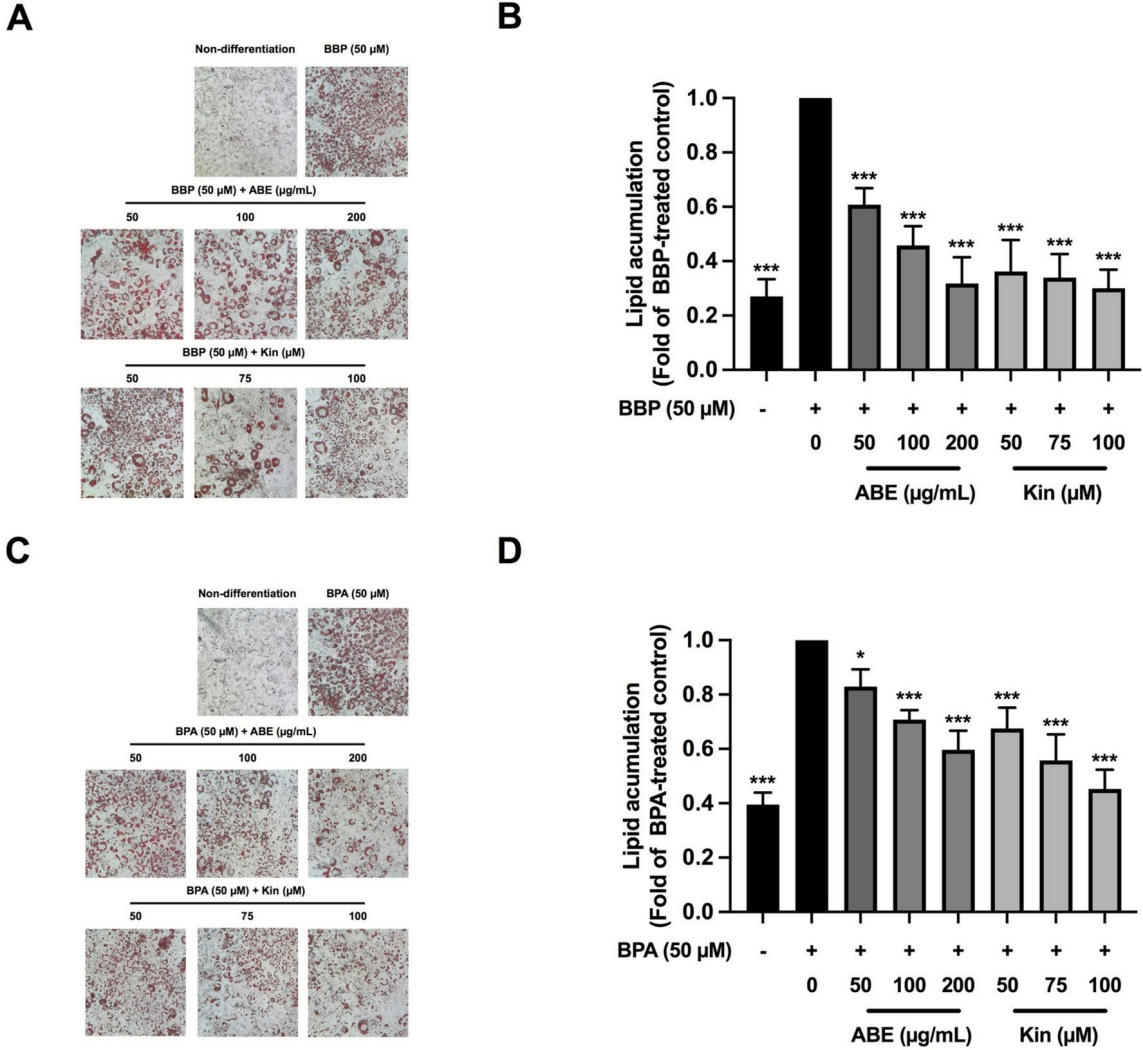
The effect of ABE or Kin on obesogen-induced lipid accumulation in 3T3-L1 cells determined by Oil-red-O staining assay. Intracellular lipid droplet (**A**), and the level of lipid accumulation (**B**) of ABE- or Kin-treated cells in the presence of BBP (50 µM). Intracellular lipid droplet (**C**), and the level of lipid accumulation (**D**) of ABE- or Kin-treated cells in the presence of BPA (50 µM). The data indicated as mean ± SD of three independent experiments *p < 0.05 and ***p < 0.001, compared to obesogen-treated control.

The original Article has been corrected.

